# Impact of Unitary Synaptic Inhibition on Spike Timing in Ventral Tegmental Area Dopamine Neurons

**DOI:** 10.1523/ENEURO.0203-24.2024

**Published:** 2024-07-19

**Authors:** Matthew H. Higgs, Michael J. Beckstead

**Affiliations:** Aging & Metabolism Research Program, Oklahoma Medical Research Foundation, Oklahoma City, OK 73104

**Keywords:** dopamine, inhibition, phase resetting, spike timing, unitary synaptic current, ventral tegmental area

## Abstract

Midbrain dopamine neurons receive convergent synaptic input from multiple brain areas, which perturbs rhythmic pacemaking to produce the complex firing patterns observed in vivo. This study investigated the impact of single and multiple inhibitory inputs on ventral tegmental area (VTA) dopamine neuron firing in mice of both sexes using novel experimental measurements and modeling. We first measured unitary inhibitory postsynaptic currents produced by single axons using both minimal electrical stimulation and minimal optical stimulation of rostromedial tegmental nucleus and ventral pallidum afferents. We next determined the phase resetting curve, the reversal potential for GABA_A_ receptor-mediated inhibitory postsynaptic currents (IPSCs), and the average interspike membrane potential trajectory during pacemaking. We combined these data in a phase oscillator model of a VTA dopamine neuron, simulating the effects of unitary inhibitory postsynaptic conductances (uIPSGs) on spike timing and rate. The effect of a uIPSG on spike timing was predicted to vary according to its timing within the interspike interval or phase. Simulations were performed to predict the pause duration resulting from the synchronous arrival of multiple uIPSGs and the changes in firing rate and regularity produced by asynchronous uIPSGs. The model data suggest that asynchronous inhibition is more effective than synchronous inhibition, because it tends to hold the neuron at membrane potentials well positive to the IPSC reversal potential. Our results indicate that small fluctuations in the inhibitory synaptic input arriving from the many afferents to each dopamine neuron are sufficient to produce highly variable firing patterns, including pauses that have been implicated in reinforcement.

## Significance Statement

This study dissects the input–output relationship of ventral tegmental area dopamine neurons receiving inputs from single presynaptic inhibitory neurons. We measured unitary inhibitory postsynaptic currents from two major inhibitory inputs (the rostromedial tegmental nucleus and ventral pallidum) and simulated their impact on dopamine neuron firing based on new experimental data including the phase resetting curve and the reversal potential of the GABA_A_ receptor-mediated currents. The results predict the impact of single and multiple unitary inhibitory postsynaptic conductances on spike timing and suggest that asynchronous, low-frequency inhibition can summate in a supralinear manner to powerfully slow or halt a dopamine neuron's firing.

## Introduction

In midbrain dopamine neurons, the firing patterns that represent established output variables such as reward prediction error emerge from a combination of intrinsic cell properties and external synaptic influences. Environmental information is conveyed to dopamine neurons through a massive number of excitatory and inhibitory afferents from many brain areas (Watabe-Uchida et al., 2012; Ogawa et al., 2014; Beier et al., 2015, 2019; Menegas et al., 2015; Faget et al., 2016; Tian et al. 2016; Dunigan et al., 2021; Beier 2022). The resulting excitatory and inhibitory postsynaptic currents are then integrated within the context of each cell's autonomous firing activity, which appears as rhythmic pacemaking in the absence of synaptic input (Sanghera et al., 1984; Trulson and Trulson, 1987; Silva and Bunney, 1988). Because synaptic input is not required for spiking, the effect of each synaptic current may be best described by the shift in spike timing that it produces. With massive input, the spike timing changes become large, producing characteristic firing patterns that include bursts and pauses (Grace and Bunney, 1984; Otomo et al., 2020).

To predict the effect of single inhibitory afferents on dopamine neurons, we first need to know the unitary synaptic conductance produced by a single spike in one axon. The gold standard for such measurements is paired recordings; however, these can be extremely difficult or impossible to obtain when the presynaptic and postsynaptic neurons are widely separated and/or pairs are connected with low probability. In some brain areas, unitary synaptic currents can be measured from spontaneous, action potential-dependent activity (Williams et al., 1998; Matsui and Williams, 2011; Higgs and Wilson, 2016; Higgs et al., 2021). In many cases, however, neither method is feasible, and unitary synaptic currents must be measured by minimal stimulation. Successful minimal stimulation is typically indicated by a mixture of zero responses (failures) and finite responses (successes) that remain consistent in size and latency over repeated threshold trials.

Because dopamine neurons are pacemakers, the next challenge in determining the fundamental effect of unitary synaptic currents is to establish their effects on spike timing. In the present study, we combined voltage- and current-clamp data to model the input sensitivity of dopamine neurons. Separate voltage- and current-clamp experiments (whole-cell and perforated-patch recording, respectively) provided ideal configurations for synaptic current and spiking measurements, and modeling allowed us to predict small changes in spike timing without requiring large numbers of trials while providing the ability to simulate arbitrary sequences of synaptic inputs.

The input sensitivity of a pacemaker neuron is best measured by the phase resetting curve (PRC), which quantifies the change in the interspike interval (ISI) produced by a brief current input as a function of the phase of the firing cycle at which the input arrives (Perkel et al., 1964; Reyes and Fetz, 1993; Stiefel and Ermentrout, 2016). We recently characterized the PRCs of substantia nigra pars compacta (SNc) dopamine neurons using noise stimuli injected via the recording electrode (Higgs et al., 2023). Here, we extended that work to describe the PRCs of ventral tegmental area (VTA) dopamine neurons, which differ somewhat in their pacemaking mechanisms (Gantz et al., 2018). The recorded neurons were located in the lateral VTA, an area including dopamine neurons that project to the lateral shell of the nucleus accumbens (Farassat et al., 2019). By combining the average interspike membrane potential trajectory with the inhibitory postsynaptic current (IPSC) reversal potential, we converted the unitary conductance waveform to a current, which shifts the timing of the succeeding action potential by an amount determined by the PRC (Simmons et al., 2018, 2020). We were then able to predict the effects of single and multiple inhibitory inputs on spike timing and rate in VTA dopamine neurons. The results suggest that the effects of individual inhibitory inputs vary according to their timing within the firing cycle and that asynchronous low-frequency inhibition can powerfully slow or halt neuronal firing.

## Materials and Methods

### Animals

All animal procedures were approved by the Oklahoma Medical Research Foundation Institutional Animal Care and Use Committee. Wild-type C57Bl/6N mice were used for all nonoptogenetic experiments. Optogenetic studies used vesicular GABA transporter (VGAT)-Cre mice (Jackson Laboratory, stock #028862). Mice of both sexes of 12 to 50 weeks old were used. Mice were bred on site, housed in a temperature-controlled facility on a 12 h light/dark cycle, and fed *ad libitum*.

### Virus injection for optogenetics

AAV5/Ef1a-DIO-hChR2-EYFP or AAV5/Ef1a-DIO-hChR2-mCherry (200–250 nl, 4 × 10^12^ viral genomes/ml; UNC Vector Core) was injected into the rostromedial tegmental nucleus (RMTg; 4.0 mm posterior, 0.5 mm lateral, and 4.7 mm ventral to the bregma) or ventral pallidum (VP; 0.5 mm anterior, 1.4 mm lateral, 5.2 mm ventral to the bregma). Experiments were performed at least 12 d after injection.

### Brain slice preparation

Mice were anesthetized deeply with isoflurane and killed by decapitation. Horizontal sections (200–220 µm) containing the ventral midbrain were prepared using a vibrating microtome (Leica VT1200S). The cutting solution was either a sucrose-based solution containing the following (in mM): 2 KCl, 1.2 NaH_2_PO_4_, 26 NaHCO_3_, 11 glucose, 250 sucrose, 7 MgCl_2_, and 0.5 CaCl_2_, or a choline chloride-based solution containing the following (in mM): 110 choline chloride, 2.5 KCl, 1.25 NaH_2_PO_4_, 0.5 CaCl_2_, 7 MgSO_4_, 25 glucose, 26 NaHCO_3_, 11.6 Na ascorbate, and 3.1 Na pyruvate. The cutting solution was bubbled with 95% O_2_, 5% CO_2_ and supplemented with 3 µM MK-801 hydrogen maleate to reduce NMDA receptor activation during slice preparation. Slicing was performed at room temperature, and the slices were then maintained at room temperature in artificial cerebrospinal fluid (ACSF) containing the following (in mM): 126 NaCl, 2.5 KCl, 1.2 MgCl_2_, 2.4 CaCl_2_, 1.2 NaH_2_PO_4_, 21.4 NaHCO_3_, and 11.1 glucose, bubbled with 95% O_2_, 5% CO_2_ and supplemented with 10 µM MK-801. Slices were allowed to recover for at least 1 h before recording and were used up to 9 h after preparation.

### Recording

**Figure 1.**
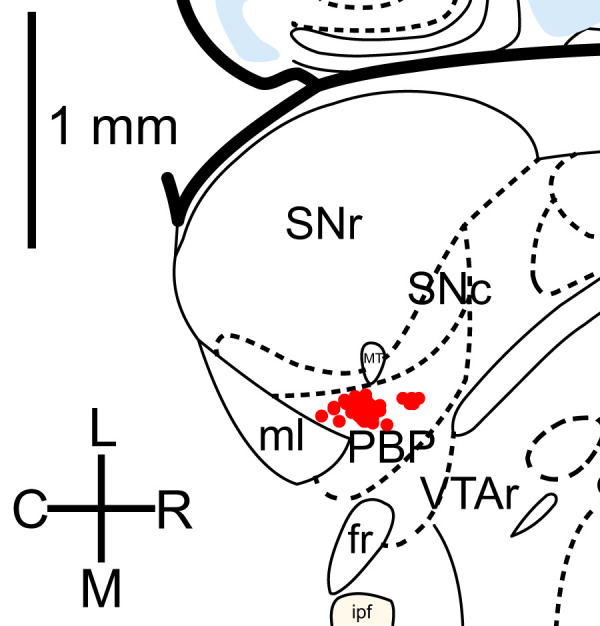
Approximate locations of recorded neurons. The figure corresponds to a horizontal section of the mouse brain 4.44 mm ventral to the bregma, adapted from Paxinos and Franklin (2019). Red symbols mark the positions of 52 recorded neurons identified photographically via a 10× objective and measured based on the mediolateral and rostrocaudal distances from the most medial point of the medial nucleus of the accessory optic tract (MT) to the tip of the recording pipette. Many of the symbols overlap in the area medial to MT. Abbreviations: SNr, substantia nigra pars reticulata; SNc, substantia nigra pars compacta; ml, medial lemniscus; PBP, parabrachial pigmented nucleus; VTAr, rostral ventral tegmental area; fr, fasciculus retroflexus; ipf, interpeduncular fossa.

Brain slices were placed on an electrophysiology rig and superfused with ACSF heated to 34°C. Neurons were visualized using a Nikon Eclipse FN1 microscope with Dodt gradient contrast optics. The recorded neurons were located in the lateral VTA ∼50–200 µm medial to the medial terminal nucleus of the accessory optic tract (MT), in the parabrachial pigmented nucleus (PBP; Fig. 1). Recordings were obtained using a MultiClamp 700B amplifier (Molecular Devices) with an ITC-18 analog/digital converter (HEKA Elektronik) controlled by AxoGraph software. Voltage-clamp data were low-pass filtered at 6 kHz, and current-clamp data were filtered at 10 kHz. All data were sampled at 20 kHz. For synaptic current recording, the ACSF contained DNQX (10 µM) to block AMPA/kainate receptors. In experiments using electrical stimulation, the ACSF also contained hexamethonium (50 µM) to block nicotinic acetylcholine receptors. IPSCs were recorded at a holding potential of −65 mV (after correction for liquid junction potential) using a pipette solution containing the following (in mM): 140.5 CsCl, 7.5 NaCl, 10 HEPES, 0.1 EGTA, 2 Mg-ATP, 0.21 Na-GTP, and 5 QX-314 Br, pH 7.0. Recording pipettes were pulled from a borosilicate glass and had resistances of ∼3 MΩ. Current-clamp recordings were obtained using the gramicidin perforated-patch method with 3–5 MΩ pipettes filled with the following (in mM): 145.5 KCl, 7.5 NaCl, 10 HEPES, pH 7.0, and 2 µg/ml gramicidin-D (MP Biomedicals). The pipette tip was filled with the same solution without gramicidin. After forming a seal on the cell membrane, perforation was allowed to proceed until overshooting action potentials were visualized and adequate bridge balance and capacitance neutralization were obtained before starting experiments. The liquid junction potential in the perforated-patch configuration cannot be calculated without knowing the exact ionic composition of the cytosol, as well as the permeability of the gramicidin pores to each ion. However, we observed that when a perforated-patch broke in suddenly, eliminating the junction potential, there was an immediate voltage change averaging +14 mV. Adding this to the liquid junction potential with the pipette in the bath (−4 mV for pipette solution vs ACSF, estimated using the LJPcalc web app), we obtained an empirical junction potential correction of +10 mV, which we added to all of the recorded voltage data. Because the same correction was applied in all of our current-clamp experiments, including the determination of GABA_A_ IPSC reversal potential, any errors in this correction did not affect our analysis of the efficacy of synaptic inhibition.

Presumptive dopamine neurons were identified by physiological criteria. In voltage-clamp experiments, whenever possible, spiking was recorded in the cell-attached configuration before breaking in, and dopamine neurons were identified by low firing rates (typically 1–3 spikes/s), highly regular ISIs, and wide action potentials. In current-clamp experiments, extensive spiking data were obtained, and additional criteria were a long, ramp-like membrane potential trajectory from the end of a hyperpolarizing current step to the first spike (rebound delay) and typical interspike membrane potential trajectories lacking a prominent fast afterhyperpolarization. In the targeted region, presumptive dopamine neurons were encountered much more frequently than presumptive GABA neurons, which were not recorded.

### Electrical stimulation

Constant-current pulses (0.1 ms, square or biphasic) were generated by a World Precision Instruments A365 stimulus isolator and delivered via a patch pipette filled with 3 M NaCl or a platinum-coated bipolar electrode (FHC) positioned at the caudal end of the VTA. For minimal stimulation, the interstimulus interval was 5 s, and the stimulus intensity was adjusted until a success (a visible synaptic current) was obtained on approximately half of the trials.

### Optical stimulation

Blue light stimuli (1 ms duration, 5 s interstimulus interval) were generated by an LED (Thorlabs SOLIS-3C) and delivered through a 445 nm excitation filter (45 nm bandwidth) and the 40× microscope objective to the channelrhodopsin (ChR2)-expressing terminal field in the VTA. For minimal stimulation, the light intensity was adjusted until a visible synaptic current was obtained on approximately half of the trials.

### Analysis of responses to minimal stimulation

Data were analyzed using custom routines written in Python. To identify single-component IPSCs of consistent latency from the stimulus, the current data were analyzed using a sensitive IPSC detection algorithm. The current trace was smoothed by convolution with a Gaussian filter (0.2 ms SD) and differentiated, and each negative peak exceeding −6 times the median absolute value of the differentiated trace was flagged as a possible IPSC onset. This algorithm can detect multiple components in many compound IPSCs (i.e., those made up of two or more uIPSCs; Fig. 4), enabling us to remove these responses from the unitary inhibitory postsynaptic current (uIPSC) data. The latencies of the detected IPSCs were plotted as a raster, and a narrow band of latencies (typically ∼1 ms in width) excluding any outliers was selected. If a narrow latency band containing the majority of the responses could not be identified, the data were rejected for the purposes of isolating a uIPSC. Trials were identified as successes if they contained a detected IPSC within the band and identified as clean (lacking any other nearby IPSCs) if they had no more than one IPSC within the band and no other IPSC within 30 ms before or after the band. The amplitudes of presumptive uIPSCs, not including failures, were determined from the clean successes. In some of the electrical stimulation experiments, the stimulus artifact was removed by subtracting the average clean failure from each response. The uIPSC time course was measured by aligning the uIPSCs at their detection points and fitting the average of the aligned responses with the sum of an exponential rising component and an exponential decay component:

(1) $$A_{\mathrm{fit}}(t)=-Ae^{-t/\tau_{\mathrm{rise}}}+Ae^{-t/\tau_{\mathrm{decay}}}.$$

### Detection and measurement of spontaneous IPSCs (sIPSCs) and evoked asynchronous IPSCs (aIPSCs)

The current trace was smoothed by convolution with a Gaussian waveform (0.3 ms SD) and differentiated, the detection level was set at −6 times the median absolute value of the differentiated trace, and an IPSC time was taken at each negative peak exceeding the detection level. To measure each IPSC, the baseline was taken as the average value of the raw current trace from −2 to −1 ms relative to the detection time. The peak was then measured as the minimum of the smoothed current trace from 0 to 2 ms after the detection time, and the difference was taken as the IPSC amplitude. Any events with nonnegative amplitudes were removed from the list of IPSCs.

### PRC measurement

Eighty 16 s blocks of broadband current noise were injected into pacemaking neurons during perforated-patch recordings, alternating each period of noise with 4 s of baseline firing to measure the neuron's unperturbed ISIs. The noise was made up of contiguous 2 ms square current pulses with amplitudes drawn from a Gaussian distribution (mean = 0; SD = 50 pA). This stimulus produced small, bidirectional changes in the ISIs and thus was neutral regarding its application to the excitatory or inhibitory input. PRCs were determined using a multiple linear regression method (Wilson et al., 2014; Higgs et al., 2023). Briefly, each ISI was divided into 40 equal-length sections, and the integral of the noise stimulus (charge) delivered in each section was determined. For regression analysis, the independent variables were the 40 values of charge for each ISI, the dependent variable was the ISI length, and the 40 regression coefficients (slope of ISI length vs charge injected) were normalized by the mean ISI to obtain the PRC value (cycles/pA-s) at each phase.

### Average interspike membrane potential trajectory

To predict the responses to synaptic conductances using the PRC, it is necessary to know the neuron's membrane potential as a function of the phase of the spiking oscillation. For this purpose, we determined the average *V_m_* trajectory for the ISIs during the baseline periods between episodes of noise stimulation. Each ISI was sampled at 10,000 equally spaced points, and the mean *V_m_* was taken for each sample point across all ISIs to obtain the average trajectory. A grand average trajectory from a sample of neurons was used for simulations.

### Reversal potential

To estimate *E*_rev_, inhibitory postsynaptic potentials (IPSPs) were evoked by electrical stimulation during the perforated-patch recording of the spontaneous pacemaking activity. AMPA, nicotinic acetylcholine, and GABA_B_ receptor-mediated currents were blocked with DNQX (10 µM), hexamethonium (50 µM), and CGP55845 (100 nM), respectively. Single stimuli were applied at 5 s intervals. Each stimulus was a charge-balanced biphasic pulse, 0.1 ms at each polarity, to eliminate the possibility of directly charging the postsynaptic membrane. To measure the IPSP slope (d*V_m_*/dt), the voltage trace was smoothed by convolution with a Gaussian function (SD = 0.5 ms) and differentiated, and the slope of each IPSP was taken at the point where the slope of the average IPSP, excluding any depolarizing responses, was most negative. The slope of the 2 ms section of the membrane potential trace immediately before the stimulus was subtracted to obtain the change in slope associated with each IPSP. The IPSP slopes were then plotted as a function of the corresponding membrane potentials, and *E*_rev_ was taken as the *x*-intercept of a linear fit to these data. Because the IPSP slopes were often observed to vary nonlinearly at the most depolarized interspike voltages, the fits were limited to a maximum voltage of −60 mV or the 75th percentile of the data, whichever was higher.

### Phase oscillator model

We simulated the spike time responses of a VTA dopamine neuron receiving synaptic conductance input using the methods described by Simmons et al. (2018). Briefly, the neuron was represented by an oscillator with a time-varying phase, *ϕ*(*t*); a natural oscillation frequency, *ω*; a synaptic conductance input, *G*(*t*); a synaptic reversal potential, *E*_rev_; a membrane potential expressed as a function of phase, *V*(*ϕ*); and a PRC, *Z*(*ϕ*). The phase is a circular variable (0 ≤ *ϕ *< 1) representing the neuron's position in the firing cycle, where the spiking point is phase 0. Thus, a spike time was recorded each time *ϕ* crossed from 1 to 0. Backward crossings from *ϕ *= 0 were not allowed. Between spikes, the phase advanced according to the following equation:

(2) $$\frac{d\phi }{dt}=\omega +G(t)[E_{\mathrm{rev}}-V(\phi )]Z(\phi ).$$

The PRC function, *Z*(*ϕ*), was obtained by fitting all but the last point of the average VTA dopamine neuron PRC with a smooth curve represented by the sum of a gamma distribution and a line:

(3) $$Z_{\mathrm{fit}}(\phi )=a\;\exp\left(-\frac{\phi -\phi_{0}}{\beta }\right)(\phi -\phi_{0}{)}^{\alpha -1}+k(\phi -\phi_{0}).$$

The fit parameters were *a *= 0.5921, *ϕ*_0 _= 0.006, *β *= 0.1128, *α *= 1.668, and *k *= 0.05637.

This function has no theoretical basis but provided a good fit to the average experimental PRC. The shape of the final, sharp peak of the PRC is not well resolved by the experimental data. In the model, the final peak was represented by a linear segment connecting the fitted curve at the second-to-last data point (*ϕ *= 0.9625) to the last (peak) data point (*ϕ*_peak _= 0.9875; *Z*_peak _= 0.1834), followed by a second linear segment connecting the peak point to a value of zero at *ϕ *= 0.999. The final segment of the PRC (0.999 < *ϕ *< 1) and the initial segment (0 < *ϕ *< 0.006) were set to zero to represent the insensitivity to synaptic input during the action potential. Simulations were performed by the first-order forward Euler integration with a time step of 0.1–0.2 ms.

### Materials

Chemicals and pharmacological agents were obtained from Sigma-Aldrich or Tocris unless indicated otherwise.

### Experimental design and statistical analysis

Two-group comparisons were performed with a Mann–Whitney U test for unpaired data or a Wilcoxon matched-pairs signed rank test for paired data. The significance of multiple-group comparisons was evaluated by a Kruskal–Wallis test, followed by post hoc Dunn's tests when a significant main effect was found. Differences were considered significant when *p *< 0.05. Confidence intervals for the difference between median values are reported in Table 1.

**Table 1.** Statistical table

Data	Data structure	Type of test	95% confidence interval of difference between medians
a. uIPSC latency, RMTg versus electric	Nonparametric	Dunn’s/Mann–Whitney	0.70–2.31 ms
b. CV of uIPSC amplitude, RMTg versus electric	Nonparametric	Dunn’s/Mann–Whitney	0.036–0.255
c. CV of uIPSC amplitude, VP versus electric	Nonparametric	Dunn’s/Mann–Whitney	0.029–0.320
d. aIPSC amplitude, VP versus RMTg	Nonparametric	Mann–Whitney	2.5–35.5 pA
e. RMTg aIPSC amplitude versus sIPSC amplitude	Nonparametric	Wilcoxon matched-pairs signed rank	−23.6 to −7.4 pA

Where two test types are indicated, the first test was used to determine the *p*-value for a post hoc comparison, and the second test was used to determine the 95% confidence interval of the difference between medians.

### Code accessibility

The code/software described in the paper is freely available online at https://github.com/higgsm/unitary-ipsc. The code is available as Extended Data.

## Results

### Minimal electrical stimulation of inhibitory input to VTA dopamine neurons

**Figure 2.**
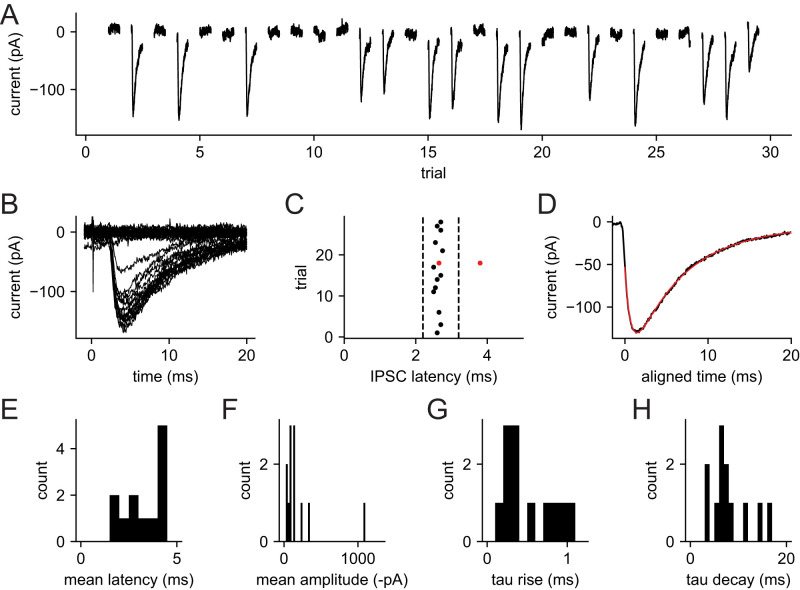
VTA dopamine neuron IPSCs evoked by minimal electrical stimulation. ***A***, Consecutive responses to a near-threshold stimulus in a single recording. The 5 s intervals between trials are omitted. ***B***, Superimposed responses. The deflection near 0 ms is the stimulus artifact. ***C***, Latency of each detected IPSC from the stimulus. Black symbols indicate the IPSC on each clean success trial, defined by a single IPSC within the indicated latency range (bracketed by dashed lines) and no other detected IPSC within 30 ms before or after that range. Red symbols indicate the IPSC times on one trial that was classified as a success based on latency but removed from the “clean success” category based on a second IPSC detected slightly later. ***D***, Average clean success IPSC in the same neuron. Each IPSC was aligned with the detection point (maximum negative slope after smoothing) at time zero before averaging. Black line is the data, and red line is a fitted curve. ***E***, Histogram of mean uIPSC latency from 12 recorded neurons. ***F***, Mean uIPSC amplitudes. ***G***, Rise time constants of the average uIPSCs. ***H***, Decay time constants of the average uIPSCs.

Voltage-clamp recordings were obtained from presumptive VTA dopamine neurons in horizontal slices. The slices were stored with MK-801 to block NMDA receptors, and AMPA/kainate and nicotinic acetylcholine receptors were blocked with DNQX and hexamethonium, respectively. All data were obtained at a holding potential of −65 mV using a high-chloride internal solution, giving a calculated chloride reversal potential of +2 mV and a driving force of −67 mV. Electrical stimuli were applied at a 5 s interval via a monopolar or bipolar electrode positioned near the caudal end of the VTA. Stimuli were carefully adjusted to near-threshold intensity, producing a mixture of successes (visible synaptic currents) and failures (Fig. 2*A,B*). All IPSCs were detected from the differentiated current trace (see Materials and Methods), their latencies were plotted as a raster, and a narrow latency band containing the most reliable responses was identified (Fig. 2*C*). Presumptive uIPSCs without contamination by other evoked or spontaneous responses were identified by a single detected event within the latency window and no other detected event (excluding the stimulus artifact) within 30 ms of either side of the window. All uIPSC measurements were obtained from these responses, which we designate as clean successes. An example of the average clean success is plotted in Figure 2*D*, and the properties of the electrically evoked uIPSCs from 12 neurons are summarized in Figure 2*E–H* and Table 2. Overall, the most striking observation from these data was the large variation of uIPSC amplitudes; most were small (<200 pA), but a few were larger, suggesting that a small fraction of the afferents might provide a substantial portion of the total inhibitory input to each neuron.

**Table 2.** Properties of uIPSCs evoked by electrical and optical stimuli

Afferents	*n*	Latency (ms)	Amplitude (successes, pA)	Amplitude CV (successes)	*τ* rise (ms)	*τ* decay (ms)
Nonspecific (electrical)	12	3.26 (SD 0.99)	−208 (SD 279)	0.23 (SD 0.13)	0.50 (SD 0.30)	8.3 (SD 4.0)
RMTg	30	4.85 (SD 1.11)	−116 (SD 124)	0.39 (SD 0.18)	0.48 (SD 0.24)	7.9 (SD 2.4)
VP	11	3.98 (SD 1.64)	−88 (SD 32)	0.42 (SD 0.18)	0.53 (SD 0.41)	6.0 (SD 2.3)

The data reported are the mean and standard deviation. Latency was measured from the onset of the electrical or optical stimulus. Amplitudes are the means for individual success responses. The time constants (*τ* rise and *τ* decay) were obtained by fitting the average success responses with the sum of a rising exponential and a single decaying exponential.

### Optically evoked unitary IPSCs

**Figure 3.**
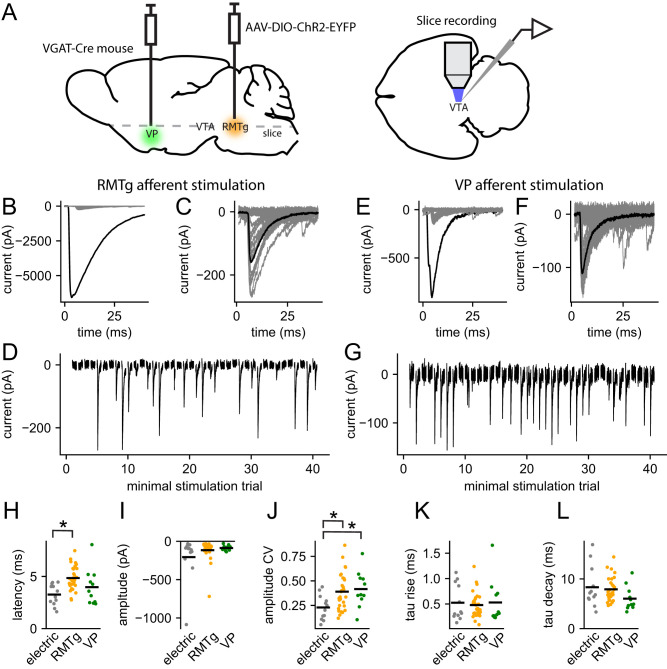
Unitary IPSCs evoked by optical stimulation of RMTg and VP afferents. ***A***, Schematic of experimental design. A Cre-dependent AAV encoding ChR2 was injected into RMTg or VP in VGAT-Cre mice. VTA dopamine neurons were recorded in horizontal brain slices, stimulating ChR2-expressing afferents with blue light flashes. ***B***, Example of maximal and minimal IPSCs from RMTg afferents in a single cell. Black trace is a response to a 1 ms light pulse of maximal intensity. Gray traces are responses to light pulses of near-threshold intensity (minimal stimulation). ***C***, Superimposed responses to minimal stimulation (gray traces) and the mean success (black trace). ***D***, Sequential responses to minimal stimulation, including successes and failures. ***E–G***, Same as ***B–D***, but for VP afferent stimulation. ***H***, Unitary IPSC latencies for all cells using electrical stimulation (electric) and optical stimulation of RMTg and VP afferents. Horizontal bars indicate the mean for each group. ***I***, uIPSC amplitudes. ***J***, CV of uIPSC amplitudes. ***K***, uIPSC rise time constants. ***L***, uIPSC decay time constants. *p < 0.05 (Dunn's test).

To activate GABAergic afferents to VTA dopamine neurons optically, we expressed ChR2 in neurons of the RMTg (formerly known as the tail of the VTA) or the VP by injection of a Cre-dependent AAV in VGAT-Cre mice (Fig. 3*A*). Experiments were performed at least 12 d after injection. ChR2-expressing afferents were stimulated at their terminal fields in the VTA using blue light flashes (1 ms) applied through the 40× microscope objective. Examples of maximal responses to RMTg and VP afferent stimulation are shown in Figure 3*B* and *E*, respectively. The maximal IPSCs produced by RMTg afferents were large (mean, −4,119 pA; range, −122 to −9586; *n *= 56), whereas VP afferents usually produced smaller maximal IPSCs (mean, −988 pA; range, −53 to −4216; *n *= 27). However, because of the limitations of viral opsin expression methods, comparison of these values may not be informative about the overall strength or connectivity of the afferents from these two areas.

**Figure 4.**
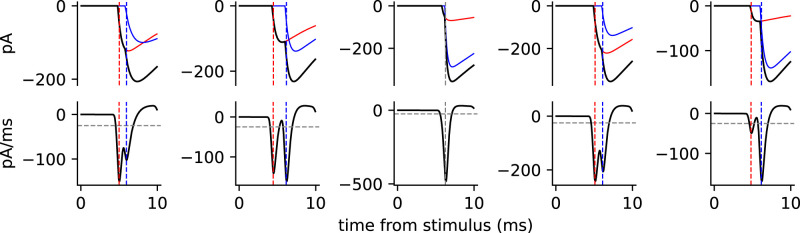
Simulation of IPSC detection for compound responses made up of two uIPSCs. Five compound IPSCs are illustrated, each based on the measured parameters for randomly chosen mean success responses to RMTg afferent stimulation. For each example, the top panel shows the compound IPSC (black line) and the two component uIPSCs (colored lines). The dashed vertical lines indicate the time where each IPSC component was detected. The bottom panels show the smoothed derivative used for IPSC detection (black line), the detection level (dashed gray horizontal line), and the detection times (dashed vertical lines). In four of the five examples, the two component uIPSCs were each detected; in the other example (middle), only one IPSC was detected. These simulations suggest that many compound IPSCs can be resolved by our analysis and removed from the presumptive uIPSC data, although some could go undetected.

When the intensity of the optical stimuli was adjusted near threshold, we could elicit a mixture of successes and failures (Fig. 3*C,D,F,G*), and from the successes, we isolated presumptive uIPSCs as described above. The properties of the uIPSCs produced by electrical stimulation and by optical stimulation of RMTg afferents (*n *= 30) and VP afferents (*n* = 11) are summarized in Figure 3*H–L* and Table 2. Overall, the optically evoked uIPSCs from these two afferent areas were similar to those evoked by electrical stimuli. Like the electrically evoked responses, the RMTg uIPSCs were mostly small but included several large responses (Fig. 3*I*). We did not obtain any large uIPSCs in the sample of neurons tested with VP stimulation, but we cannot exclude the possibility that some strong unitary VP inputs would be observed with a larger sample size. The only significant differences among the uIPSC measures obtained with the three stimulus types were for the latencies (*p *= 0.0014, Kruskal–Wallis test; electrical vs RMTg, *p *= 0.0018^a^, Dunn's test) and the coefficient of variation (CV) of success amplitudes within each cell, which was higher for both optical stimulation groups compared with the electrical stimulation group (*p *= 0.023, Kruskal–Wallis test; electrical vs RMTg, *p *= 0.048^b^; electrical vs VP, *p *= 0.040^c^, Dunn's test). A difference in latency for optical versus electrical stimulation is not unexpected because of the relatively slow kinetics of ChR2, although this difference was not significant for VP afferent stimulation versus electrical stimulation. The basis of the larger CV of uIPSC success amplitudes in the optical stimulation groups compared with the electrical stimulation group is not known. However, in the case of RMTg input, a contributing factor could be larger quantal size (see results below).

### Could we discern compound IPSCs?

A common objection to the minimal stimulation method is that some responses may be compound synaptic currents made up of more than one unitary component, if two or more stimulated axons produce synaptic currents with very similar latencies. To estimate the likelihood that such compound responses would go undetected, we constructed 435 simulated compound IPSCs, each with two unitary components, and ran them through our detection algorithm to determine whether two components would be detected. Each uIPSC in a simulated compound IPSC had the latency, amplitude, rise time constant, and decay time constant of one of the mean presumptive uIPSCs in the RMTg sample described above. Figure 4 shows five of these simulated compound IPSCs and their component uIPSCs, along with the filtered derivative used for IPSC detection. An IPSC was detected at each negative peak in the filtered derivative that exceeded the detection level, which was determined based on the noise level in each smoothed, differentiated current trace (see Materials and Methods). The average detection level determined for the experimental data was ∼25 mV/ms, and this value was used in the simulations. We found that two IPSCs were detected in 297 of the 435 compound IPSCs, or ∼68%. This result indicates that if any compound IPSCs are evoked by our near-threshold stimuli, a majority of them should be rejected by our analysis. However, some compound IPSCs could escape detection, particularly when the two latencies are very similar and/or one of the uIPSCs is much larger than the other. This possibility becomes less likely when the data include a high proportion of failures; in this case, we would expect to observe uIPSCs from the two afferents separately on some trials, likely producing recognizable differences in uIPSC amplitudes and/or kinetics that allow rejection of responses from more than one axon. In our data for RMTg afferent stimulation, the mean failure probability was 0.49 (SD, 0.19). Indications of more than one stimulated afferent were indeed observed in some experiments, and in these cases, the data were rejected. With these precautions taken, none of our three datasets showed a significant correlation between the success probability and the mean uIPSC amplitude across neurons, suggesting that higher-probability responses did not usually hide multiple components. For these reasons, we believe that compound responses were not prevalent in our accepted data.

### Quantal responses from RMTg and VP afferents

**Figure 5.**
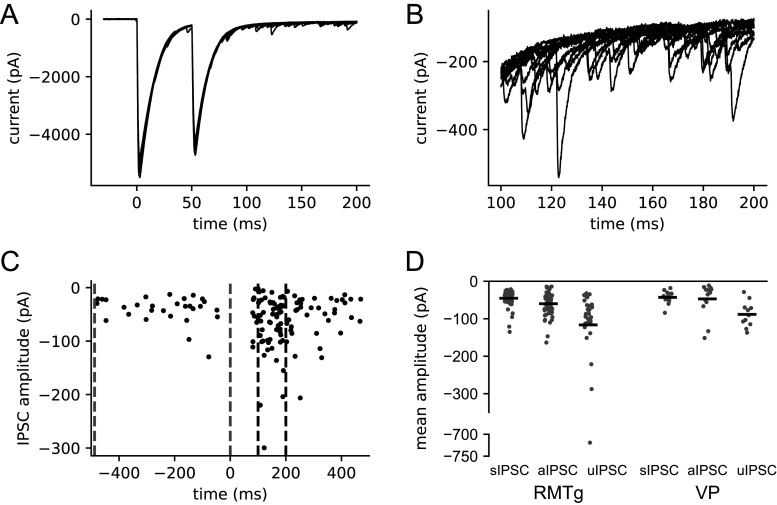
aIPSCs after strong optical stimulation. ***A***, Example of IPSCs evoked by paired 1 ms light pulses of maximal intensity delivered to ChR2-expressing RMTg afferents. Ten trials are superimposed. ***B***, Expanded view of the same traces from 50 to 150 ms after the second stimulus. ***C***, Amplitudes of the detected IPSCs in the example traces, excluding the synchronous evoked IPSCs. Gray dashed lines bracket the time range for the measurement of baseline sIPSCs, and black dashed lines indicate the time range for the measurement of evoked aIPSCs. ***D***, Mean amplitudes of sIPSCs, aIPSCs, and uIPSCs in each cell tested with RMTg or VP afferent stimulation.

Because single axons often form multiple synapses on a postsynaptic neuron, many uIPSCs are likely produced by the release of more than one neurotransmitter vesicle, or quantum. To estimate how many quanta contribute to uIPSCs, we measured the amplitudes of aIPSCs that occur with elevated frequency after large evoked IPSCs. These presumably quantal events were most prominent after strong optical stimulation of RMTg afferents (Fig. 5*A,B*) and were also observed in most experiments with strong stimulation of VP afferents. We measured the amplitudes of aIPSCs in a window from 50 to 150 ms after single or paired stimulation (Fig. 5*C*). To account for the ongoing sIPSCs not evoked by the stimuli, we also measured the rate and amplitudes of sIPSCs in the baseline period before stimulation. The mean amplitude of evoked aIPSCs (*μ_A_*) was estimated as follows:

(4) $$\mu_{A}=\frac{r_{\mathrm{post}}\mu_{\mathrm{post}}-r_{\mathrm{pre}}\mu_{\mathrm{pre}}}{r_{\mathrm{post}}-r_{\mathrm{pre}}},$$

where *r*_pre_ and *r*_post_ are the IPSC rates before the stimulus and in the poststimulus window, respectively, and *μ*_pre_ and *μ*_post_ are the mean IPSC amplitudes in these periods. The aIPSCs evoked by RMTg afferent stimulation had an overall mean amplitude of −59.9 pA (range, −14.1 to −163.5; *n *= 56), whereas those produced by VP afferent stimulation had a smaller overall mean amplitude of −47.2 pA (range, −11.3 to −151.5; *n *= 14; *p *= 0.03^d^, Mann–Whitney test). RMTg aIPSCs were larger than the prestimulus sIPSCs (mean, −45.4 pA; range, −21.4 to −135.1) (*p *< 0.0001^e^, Wilcoxon matched-pairs signed rank test), whereas VP aIPSCs did not differ significantly from the baseline sIPSCs. Figure 5*D* compares the mean amplitudes of sIPSCs with those of aIPSCs and uIPSCs produced by RMTg and VP afferent stimulation. For RMTg the mean uIPSC was 1.94 times the mean aIPSC, and for VP the mean uIPSC was 1.87 times the mean aIPSC. These data suggest that afferents from both sources release a mean quantal content of ∼2 under our experimental conditions, although many of the smaller uIPSCs were in the range of single quanta.

### IPSP reversal potential

**Figure 6.**
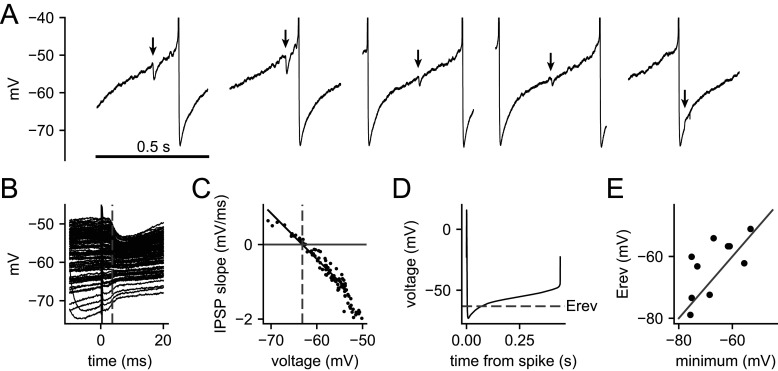
IPSP reversal potential estimated by electrical stimulation during pacemaker firing. ***A***, Example of IPSPs evoked at different times within the ISI, each indicated by an arrow. The stimulus artifacts are blanked. ***B***, Expanded view of the responses to 100 stimuli in the same cell. The deflections immediately after 0 ms are the stimulus artifact. The dashed gray line indicates where the slope of each response (IPSP slope) was measured, subtracting the prestimulus slope. ***C***, IPSP slope versus membrane potential taken at the same point. Black line is a linear fit to the data. Data points beyond the right end of the fit line were not fitted. Dashed gray line indicates the measured reversal potential, taken as the *x*-intercept of the fit. ***D***, Average prestimulus interspike membrane potential trajectory of the example cell. The dashed gray line indicates the IPSP reversal potential (*E*_rev_). ***E***, *E*_rev_ values from 10 VTA dopamine neurons, plotted versus the minimum of the average interspike membrane potential trajectory for each cell. In seven of ten neurons, *E*_rev_ was above the minimum of the trajectory, indicating that during pacemaker firing, GABAergic input was excitatory early in the ISI.

To determine the impact of uIPSCs on a postsynaptic neuron, it was necessary to measure the reversal potential (*E*_rev_) for inhibitory input to VTA dopamine neurons. Previous reports showed that SNc dopamine neurons in mature rodents had GABA_A_ IPSP/IPSC reversal potentials ranging from a value of −63 mV obtained using the gramicidin perforated-patch method (Gulácsi et al., 2003) to values of −79 to −68 mV obtained by sharp electrode intracellular recording (Grace and Bunney, 1985; Johnson and North, 1992; Häusser and Yung, 1994). To estimate *E*_rev_ under the same conditions used for our spiking experiments (see the following sections), we measured electrically evoked IPSPs during pacemaker firing, obtained during gramicidin perforated-patch recordings while blocking AMPA/kainate, nicotinic acetylcholine, and GABA_B_ receptor-mediated responses. Because the stimuli arrived at various times within the ISI, we were able to sample a range of membrane potentials without injecting current, eliminating the risk of voltage errors caused by imperfect bridge balance. Example responses are shown in Figure 6*A* and *B*. The membrane potential responses occurred with a short latency after the stimulus artifact, which did not appear to disturb the trajectory prior to the IPSP onset. In the example, most of the responses were hyperpolarizing, but those starting from the most hyperpolarized potentials (early in the ISI) were depolarizing. The slope of each response (IPSP slope) was measured at the point where the slope of the average hyperpolarizing IPSP was maximal, which generally occurred shortly after the IPSP onset (Fig. 6*B*, dashed gray line). The slope immediately before the stimulus was subtracted to obtain the change in slope associated with the IPSP. Each IPSP slope was plotted against the corresponding membrane potential, taken at the same point (Fig. 6*C*), and *E*_rev_ was determined as the *x*-intercept of a linear fit, excluding points at the most depolarized voltages, where the relationship often became nonlinear (see Materials and Methods). In 10 VTA dopamine neurons, the mean *E*_rev_ was −63 mV (range, −79 to −51 mV). A key factor determining the functional effect of the inhibitory synaptic input is the relationship between *E*_rev_ and the interspike membrane potential trajectory (Fig. 6*D*). In 7/10 cells, *E*_rev_ was positive to the minimum of the average trajectory (Fig. 6*E*), indicating that during pacemaker firing, the responses to inhibitory afferents were excitatory in the deepest portion of the afterhyperpolarization.

### Phase resetting in VTA dopamine neurons

To predict the effects of synaptic input on the spike output of VTA dopamine neurons, we first determined how these cells' input sensitivity varies across each ISI. This information is provided by the PRC, which quantifies the change in the ISI length produced by a given charge injection as a function of the phase of the ISI at which the charge is delivered. We measured VTA dopamine neuron PRCs in perforated-patch mode using noise current injected via the recording electrode. We recently reported similar measurements from SNc dopamine neurons (Higgs et al., 2023). However, VTA dopamine neurons are known to differ from SNc neurons in their pacemaking mechanisms, having larger voltage-gated and leak sodium currents, smaller L-type calcium and HCN currents, and slower inactivation of the A-type potassium current (Khaliq and Bean, 2010; Philippart et al., 2016; Tarfa et al., 2017; Cobb-Lewis et al., 2023; reviewed by Gantz et al., 2018). Thus, their PRCs could be different.

**Figure 7.**
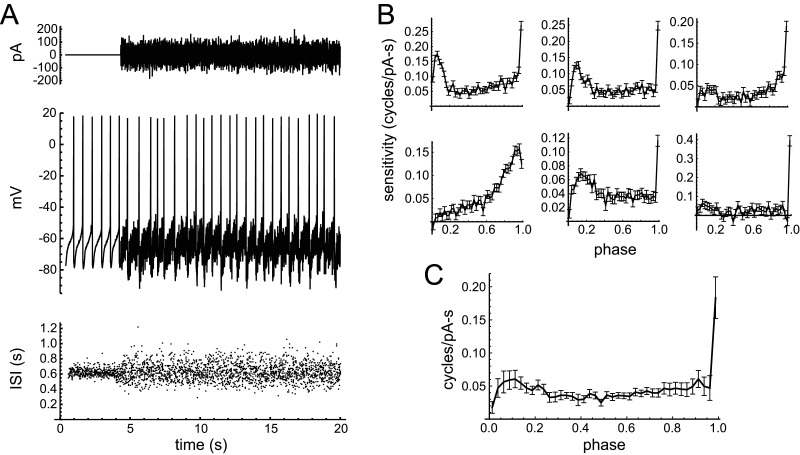
PRCs of VTA dopamine neurons. ***A***, Example of the noise stimulus (top), the perturbed spiking (middle), and the ISIs for 80 trials (bottom), each including 4 s of baseline firing followed by 16 s of noise injection. The zero-mean noise stimulus perturbed the ISIs bidirectionally, with relatively little effect on the mean firing rate. ***B***, PRCs of six VTA dopamine neurons, with the example neuron's PRC at the upper left. The PRC data indicate each cell's sensitivity, in terms of the change in the ISI length per unit of charge injected, at 40 phases spanning the ISI. Error bars indicate the standard error of the estimate for each cell at each phase. The PRCs vary among individual neurons, but most have a broad body showing sensitivity across most of the ISI, followed by a sharp peak indicating increased sensitivity close to the firing point. ***C***, Average of 10 VTA dopamine neuron PRCs. Error bars indicate the SEM across cells at each phase.

The noise stimulation experiment used for PRC estimation is illustrated in Figure 7*A*. Because the injected current had a mean of zero, its major effect was to increase the variance of ISIs, with relatively little effect on mean firing rate. The PRC was calculated from the noise waveform and the spike times using a multiple regression method (Wilson et al., 2014) in which each ISI was divided into 40 equal-length sections, the charge injected in each section was measured, and the slope of the relationship between the injected charge and the ISI length was determined for each section (i.e., each phase of the ISI). The resulting PRC for the example neuron is illustrated in Figure 7*B* (top left), along with the PRCs of five other VTA dopamine neurons, and the average PRC for the sample of 10 neurons is shown in Figure 7*C*. The main features of these PRCs were a broad body that could slope either downward or upward across most of the ISI and a sharp peak at the end, indicating high sensitivity shortly before spiking. In general, these PRCs were similar to those determined previously in SNc dopamine neurons, including the final sharp peak (Higgs et al., 2023), although PRCs with the main body peaking at an early phase may be more common in the VTA neurons.

### Lack of spike frequency adaptation

**Figure 8.**
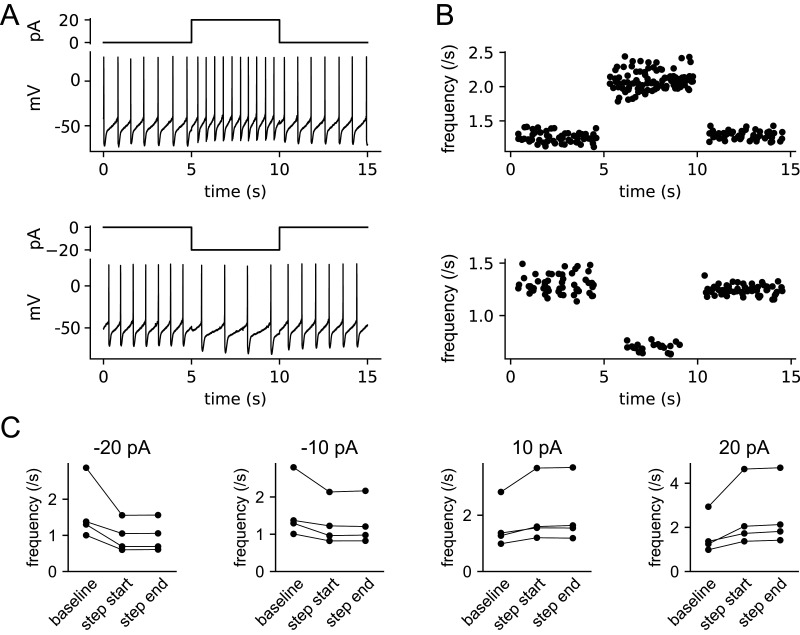
Lack of spike frequency adaptation. ***A***, Example of VTA dopamine neuron responses to +20 and −20 pA, 5 s current steps. ***B***, Firing *f*–*t* data for the example cell, including data points from 10 trials at each current level. Each frequency is the inverse of an ISI and is plotted against the time at the ISI midpoint. Data points from ISIs spanning the step onset or offset are not plotted. ***C***, Mean firing frequency of each cell at the last baseline ISI (baseline), the first ISI during the step (step start), and the last ISI during the step (step end).

In general, the PRC can predict a pacemaking neuron's response to transient input but is not sufficient to predict the response to sustained input, because it does not capture any effects that last beyond the present ISI. However, many neurons show pronounced spike frequency adaptation that accumulates over multiple ISIs, reducing their responses to sustained current or long trains of synaptic inputs. If such adaptation is present, it should be incorporated in a model used to predict a neuron's responses to long-duration inputs. To determine whether the firing frequency responses of VTA dopamine neurons adapt to small-amplitude depolarizing or hyperpolarizing inputs of long duration, we injected 5 s current steps with amplitudes from −20 to +20 pA into neurons recorded in perforated-patch, current-clamp mode. Example spike responses are shown in Figure 8*A*, and the corresponding firing frequency–time (*f*–*t*) data for 10 trials are plotted in Figure 8*B*. In each of the four neurons tested, there was no appreciable spike frequency adaptation from the start to the end of the 5 s current steps (Fig. 8*C*). Based on these observations, we simulated the responses to sustained inhibitory barrages without including any adaptation in our model (see below).

### Impact of unitary inhibitory postsynaptic conductances (uIPSGs) on firing

**Figure 9.**
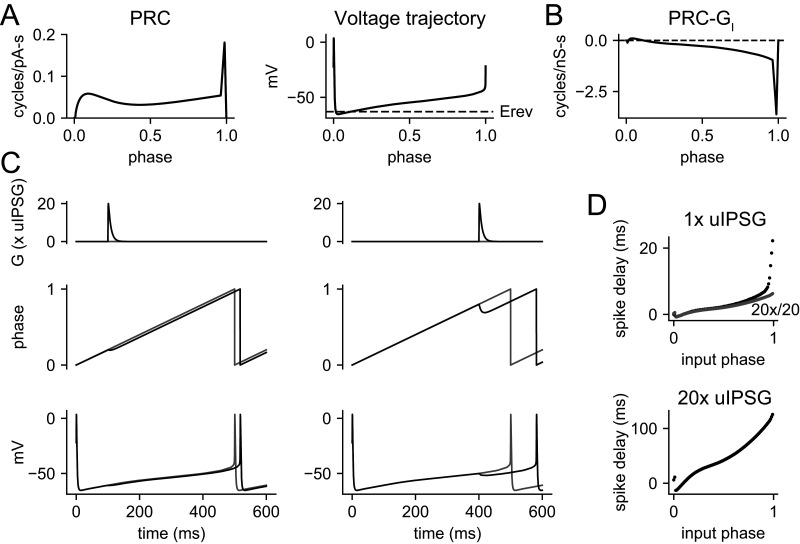
Simulated responses to unitary IPSGs and synchronous compound IPSGs. Simulations were performed by numerical integration in a phase oscillator model constructed based on our experimental data. ***A***, Components of the phase model. Left: PRC function, based on a fit to the average experimental PRC. Right: voltage trajectory represented as a function of phase, *V*(*ϕ*). Dashed line indicates the IPSC reversal potential (*E*_rev_) of −63 mV. ***B***, PRC for inhibitory conductance, given by the PRC for current, *Z*(*ϕ*), times the driving force, *E*_rev_ − *V*(*ϕ*). ***C***, Model responses to a single IPSG (20× mean uIPSG) delivered at a phase of 0.2 (left) or 0.8 (right). Top: synaptic conductance. Middle: phase trajectory. Bottom: voltage trajectory. Because the model voltage is a function of a phase, the voltage perturbation is secondary to the phase perturbation. Gray lines indicate the unperturbed trajectory, and black lines indicate the trajectory perturbed by the IPSG. ***D***, Spike time changes produced by one uIPSG (top) or 20 synchronous uIPSGs (bottom), plotted versus the phase at which the IPSG was delivered. Gray symbols in the top graph indicate the spike time changes produced by 20 uIPSGs, normalized to the change per uIPSG (20×/20). This comparison indicates that the summation of 20 uIPSGs was almost linear at most phases but became sublinear near the spike phase.

We next predicted the effects of unitary synaptic inhibition on the spike output of a typical VTA dopamine neuron using a phase oscillator model incorporating our experimental uIPSC measurements (expressed as conductance), the GABA_A_ reversal potential, the interspike membrane potential trajectory, and the PRC (Fig. 9*A*). A phase model embodies the simplifying concept that during pacemaker firing, the neuron is confined to a set of states (i.e., combinations of state variables) that form a limit cycle in state space. With this assumption, the large number of possible states that could describe the neuron at any moment can be reduced to a single state variable, the phase, (*ϕ*), that identifies the neuron's position along the firing cycle (Stiefel and Ermentrout, 2016). Our problem in simulating the neuron's input–output relationship is then reduced to determining the phase change produced by a given synaptic input, which is accomplished by integrating a phase equation (see Materials and Methods; Eq. 2), in which the external current alters the rate of phase advance with a sensitivity given by the PRC value at the neuron's present phase. The PRC function used in the model was a curve fitted to the mean experimental PRC, with a final triangular peak added to account for the high value of the last experimental data point. To simulate the response to a synaptic conductance, the model also needs a voltage variable to determine the driving force for the synaptic current. For this purpose, we used the experimental average interspike membrane potential trajectory obtained from the neurons used to measure *E*_rev_. In a phase model, every state variable must be a function of phase, and thus, the voltage trajectory was expressed as a function of phase, *V*(*ϕ*). The product of the PRC, *Z*(*ϕ*), and the driving force, *E*_rev_ − *V*(*ϕ*), is the PRC for inhibitory conductance (PRC-G_I_; Fig. 9*B*), which demonstrates how the sensitivity to synaptic inhibition varies across the firing cycle.

Using this model, we first simulated the effects of a single IPSG arriving at a specified phase of the ISI. All simulations were performed with a natural oscillation frequency (pacemaking rate) of 2 s^−1^, which is close to the average firing rate from our recordings. The input to the model is a synaptic conductance waveform, *G*_syn_(*t*), and the output is the time-varying phase of the neuron, *ϕ*(*t*). The spike times are taken where *ϕ*(*t*) reaches the end of each cycle, wrapping around from 1 to 0. The IPSG amplitude was chosen as a multiple of the mean experimental uIPSG for RMTg input (peak amplitude = 1.731 nS; rise time constant = 0.5 ms; decay time constant = 7.9 ms). In a noiseless phase model without synaptic input, *ϕ*(*t*) advances monotonically at the natural oscillation rate, generating spikes at regular intervals. The arrival of an IPSG alters the rate of phase advance with an efficacy given by PRC-G_I_, so an IPSG delivered at an early phase produces a much smaller phase change compared with the one delivered later in the firing cycle (Fig. 9*C,D*). On average, a single uIPSG delayed the next spike by 3.26 ms, or 0.65% of a cycle. The delay varied drastically as a function of the input phase, from a phase advance of 0.14% of a cycle for a uIPSG arriving at a phase of 0.01 to a phase delay of 4.44% of a cycle for a uIPSG delivered at a phase of 0.99.

**Figure 10.**
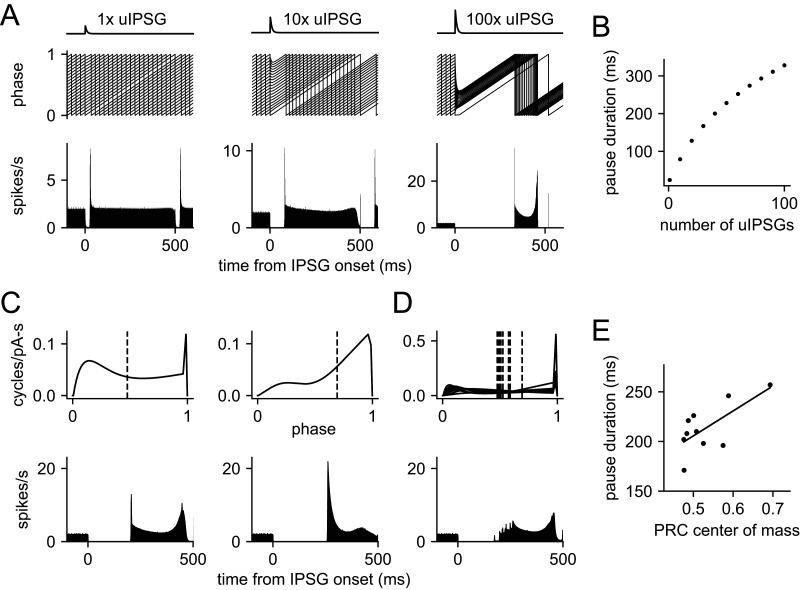
Simulated PSTH for synchronous inhibition. ***A***, PSTH for 1, 10, and 100 synchronous uIPSGs. Top: the IPSG waveform (not to scale vertically). Middle: sample phase trajectories for simulations starting at phases between 0 and 1. Bottom: PSTH obtained from simulations starting at 10,000 phases uniformly distributed from 0 to 1. ***B***, Pause duration produced by 1–100 synchronous uIPSGs. The pause can be much longer than the IPSG, but increases in a sublinear manner with the number of uIPSGs. ***C***, Effect of PRC shape heterogeneity on the PSTH produced by 50 synchronous uIPSGs. Top: PRC functions for two single VTA dopamine neurons, obtained by fitting the PRC data from each cell with Equation 3. Each PRC function was normalized to the same mean amplitude as the sample average PRC. Dashed vertical lines indicate the center of mass of each PRC. Bottom: simulated PSTH for 50 synchronous uIPSGs obtained with each PRC. ***D***, Top: normalized PRC functions of 10 VTA dopamine neurons. Bottom: average PSTH in response to 50 synchronous uIPSGs using the 10 normalized PRC functions. ***E***, Single-neuron PSTH pause duration versus PRC center of mass for the 10 normalized PRCs. Line is a linear fit (*r *= 0.69; *p *= 0.03, correlation test).

If we apply an IPSG of a given size at phases uniformly distributed between 0 and 1, we can generate a poststimulus time histogram (PSTH; Fig. 10*A*). The PSTH shows a pause beginning shortly after the IPSG onset, because trajectories approaching the firing point (Phase 1) are pushed back strongly by the inhibitory conductance. The pause duration does not directly reflect the time course of the IPSG, but instead grows as a function of the IPSG amplitude (Fig. 10*B*), because of the increasing perturbation of the neuron's phase. A single uIPSG caused a pause of ∼13 ms, or 2.6% of the natural ISI. Comparing this value with the mean phase delay caused by one uIPSG, we see that the pause was greater, as it depends on the late-phase sensitivity of the neuron rather than the average sensitivity. Increasing numbers of synchronous uIPSGs lengthened the pause in a sublinear manner. The sublinearity arises because large, synchronous IPSGs drive the phase back to where PRC-G_I_ is small. Thus, the phase change caused by a large IPSG reduces the neuron's sensitivity during the time course of the IPSG decay.

The simulations described so far all used the sample average PRC to represent a typical VTA dopamine neuron. However, the PRCs of individual cells varied in their average amplitude and shape. The effect of scaling the PRC is equivalent to the same scaling of the input amplitude and is therefore predictable from the results described above, but the effects of the PRC shape are less obvious. To investigate these, we constructed a PRC function from the PRC data obtained in each of the 10 neurons in our sample, normalizing each PRC function to the mean amplitude of the sample average. We then simulated the PSTH for 50 synchronous uIPSGs as described above (Fig. 10*C*). The results indicate that the PRC shape can influence the PSTH, even when the mean PRC amplitudes are matched. In general, PRCs with a higher center-of-mass phase produced longer pauses (Fig. 10*E*), consistent with the idea that pause duration is governed primarily by the neuron's sensitivity in the later portion of the ISI. Variations in the PRC shape also affected the form of the PSTH after the pause. As a result, the simulated population PSTH for our sample of VTA dopamine neurons (Fig. 10*D*) showed a more gradual resumption of firing after the pause and lacked the sharp peaks obtained in simulations with a single PRC.

### Predicted effects of asynchronous uIPSGs

**Figure 11.**
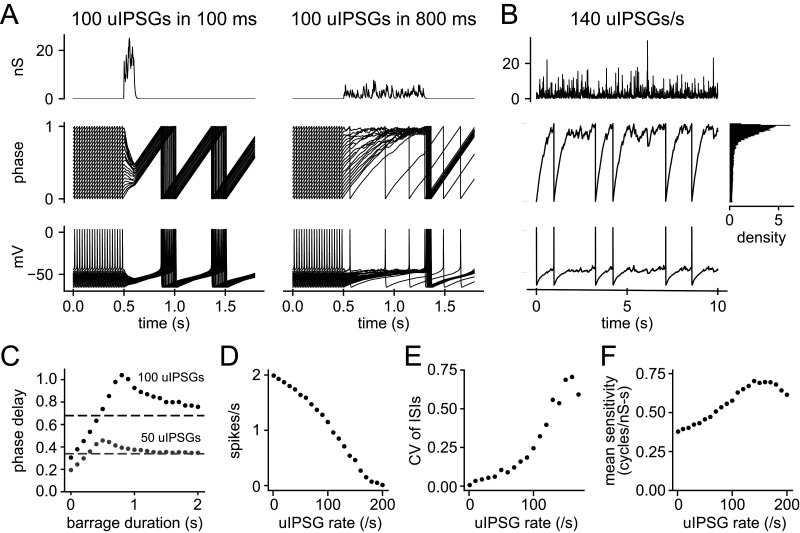
Simulated responses to asynchronous uIPSG barrages. The indicated numbers or rates of uIPSGs were applied with random timing, as a Poisson process. ***A***, Top: example of the applied conductance waveforms for 100 uIPSGs delivered in a 100 ms window (left) and an 800 ms window (right). Middle: phase trajectories for 20 trials, each starting with the neuron at a different phase and receiving uIPSGs with new random timing. Bottom: the corresponding voltage trajectories. ***B***, Same as ***A***, but for a continuous barrage of 140 uIPSGs per second (one trial, total duration 100 s). Marginal density plot at the right indicates the probability density of phase during the barrage, showing that the model neuron had a high probability of being at a late phase as it received the inhibitory barrage. ***C***, Mean phase delay caused by 50 or 100 uIPSGs delivered over durations from 0 s (synchronous input) to 2 s. The dashed gray and black lines indicate 50 and 100 times the mean delay caused by a single uIPSG, the predictions for linear summation. When the uIPSGs were applied in a short duration, the summation was sublinear, with the delay falling below the dashed line. When barrages containing the same number of uIPSGs were spread over longer durations, the summation was supralinear. ***D***, Mean firing rate versus steady-state uIPSG rate, determined with 100 s barrages. ***E***, CV of ISIs versus steady-state uIPSG rate. ***F***, Mean sensitivity to inhibitory conductance input at each steady-state uIPSG rate, given by the mean value of PRC-G_I_(*ϕ*) during the barrage.

Highly synchronous synaptic inputs are not typical in vivo, so we performed additional simulations to predict the effects of uIPSGs arriving in more physiological asynchronous barrages. We first compared barrages containing a fixed number of uIPSGs, each with the amplitude and time course described above, but distributed over narrow or wide time windows. The uIPSG arrival times were randomized on each trial, with a uniform probability across the window. Examples of the barrages, the resulting phase trajectories, and the corresponding voltage trajectories are shown in Figure 11*A*. To determine the effects of steady-state inhibition, we applied 100 s barrages of asynchronous uIPSGs to the model neuron (Fig. 11*B*). These steady-state simulations also accounted for the experimentally characterized variability of uIPSG amplitudes by drawing each amplitude from a gamma distribution with the experimental mean and SD for a randomly selected RMTg uIPSG. Because of the lack of substantial spike frequency adaptation in our recorded neurons, the steady-state simulations should predict the responses to natural inputs up to at least several seconds in duration.

For the nonsteady-state barrages, we quantified the net inhibition produced as the average phase delay compared with the free-running model with no input, collecting the final phase at the end of each trial and adding the spike count to remove the effect of each reset from Phase 1 to 0 (i.e., to obtain the final value of the unwrapped phase). We compared these phase delays to the linear prediction given by the delay produced by a single uIPSG times the number of uIPSGs in the barrage (Fig. 11*C*). As demonstrated above, the summation of synchronous uIPSGs (zero barrage duration) was sublinear, producing a delay less than the linear prediction. However, with increased barrage duration, the summation became supralinear. A maximum average delay was obtained with a barrage length of ∼800 ms for 100 uIPSGs and ∼500 ms for 50 uIPSGs. When the same numbers of uIPSGs were spread over longer windows, the average phase delay decreased, approaching the linear prediction. Examining the phase trajectories, we see that the barrage length producing the greatest phase delay caused the phase trajectories to converge at a late phase, from which occasional spikes were generated. As described above, late phase is where synaptic inhibition is most effective, which explains the supralinear summation of phase delays.

For steady-state (100 s) barrages, we measured the mean firing rates (Fig. 11*D*) and the CV of the ISIs (Fig. 11*E*), plotting each as a function of the rate of applied uIPSGs. Increasing uIPSG rates reduced the average firing rate of the model neuron in a supralinear manner, before approaching the floor of no firing, and also had a supralinear effect on the CV. The supralinearity can be explained by the steady-state distribution of phase during the barrage (Fig. 11*B*, inset, marginal distribution of phase). At a sufficiently high uIPSG rate, the model neuron spent a large fraction of the time at late phases, where the sensitivity to inhibition is high. We quantified the average steady-state sensitivity by determining the mean value of PRC-G_I_ during each barrage (Fig. 11*F*). The average sensitivity increased with the uIPSG rate up to ∼140 uIPSGs/s, at which point the average firing rate was about a quarter of the unperturbed rate and the ISI variability was high. These results confirm that individually small inhibitory inputs distributed over time can increase one another's efficacy by causing the neuron to stall at a late phase of the firing cycle. Similar effects were reported previously for putative GABA neurons in the substantia nigra pars reticulata (Simmons et al., 2020). These results predict that even sparse inhibition from a large number of inhibitory afferents will powerfully shape the dopamine neuron's spike output.

## Discussion

We characterized uIPSCs produced by RMTg and VP afferents to VTA dopamine neurons, and by local electrical stimulation, and predicted their effects on the spike output of VTA dopamine neurons. Unitary IPSCs from these three sources were similar, although RMTg might provide more strong inputs compared with VP. To predict the impact of inhibitory afferents on firing, we determined the PRCs of a sample of neurons, the IPSC reversal potential, and the mean interspike membrane potential trajectory. Combining these data in a phase oscillator model, we simulated the responses to synchronous and asynchronous uIPSGs. The results suggest that the spike time change produced by each uIPSG is small, but summation of asynchronous inhibition can be supralinear, potentially allowing small changes in inhibitory afferent activity to slow or halt dopamine neuron firing.

Consistent with this powerful effect, GABAergic inhibition is critical for dopamine neurons' responses to behaviorally relevant events including aversive stimuli (Jhou et al., 2009; Li et al., 2019; Liu et al., 2022; Du et al., 2023; Brown et al., 2024) and reward prediction errors (Hong et al., 2011; Chang et al., 2016; Sharpe et al., 2017). The phase resetting mechanism may allow inhibition associated with reward expectation to have a subtractive effect on the dopamine neuron's output firing rate (Eshel et al., 2015), by translating inhibitory barrages into a slowed rate of phase advance. However, detailed predictions of phase models under such conditions will require experimental validation.

### Technical considerations

In an attempt to isolate clean uIPSCs, we used near-threshold stimulation, adjusting stimulus intensity to produce a mixture of successes and failures. The main concerns with this approach are that some successes might include more than one uIPSC and that even true uIPSCs might arrive from different afferents on successive trials. To minimize contamination of this kind, we selected a narrow range of response latencies and rejected detected multicomponent responses. Different unitary inputs here had a range of different latencies, as reported from the earliest studies of unitary synaptic responses (Eccles et al., 1966). Based on measured latencies and other uIPSC parameters for RMTg input, our simulations predict that ∼68% of two-uIPSC responses could be resolved by our detection algorithm, substantially reducing the chance of erroneously including compound responses. Given this, in addition to other measures employed, we believe it is highly unlikely that dual uIPSCs substantially contaminated the dataset or affected our results. Another potential issue with minimal stimulation is that the axons most easily stimulated might not represent an unbiased sample of the afferents from a given source. To evaluate this, methods would need to be developed to analyze the unitary components of compound responses, to achieve more asynchronous stimulation of different axons, and/or to maintain intact connections producing naturally asynchronous synaptic responses (Matsui and Williams, 2011; Higgs and Wilson, 2016; Jones et al., 2024).

### Unitary IPSC properties

Comparing our measured uIPSCs with asynchronous quantal IPSCs, the average uIPSCs represented ∼2 quanta. This is likely a slight overestimate, because our minimal stimulation method did not distinguish stimulation failures (no presynaptic action potential) from synaptic failures (action potential but no transmitter release). If synaptic failures could be included in the data, the average uIPSC amplitudes would be somewhat lower. The small quantal content of most uIPSCs suggests that, assuming the average release probability was at least 0.2 under our experimental conditions, a typical inhibitory afferent may form <10 synapses on each dopamine neuron. To our knowledge, the total number of GABAergic synapses on VTA dopamine neurons has not been determined. However, SNc dopamine neurons were reported to receive ∼4,000 GABAergic boutons (Henny et al., 2012). If this number is similar for VTA dopamine neurons, each one may receive on the order of 400–1,000 GABAergic afferents.

Although the afferents to dopamine neurons are numerous, they are not all equal in strength. Most uIPSCs were small, but a few were much larger. In addition, while adjusting stimuli to obtain minimal responses, we often observed large jumps in IPSC amplitude at higher stimulus intensities, suggesting that many VTA dopamine neurons receive at least one strong inhibitory input. Long-tailed distributions of unitary synaptic strength have been observed previously in other neuron types (Sayer et al., 1990; Maccaferri et al., 2000; Higgs and Wilson, 2016; Guan et al., 2017; Higgs et al., 2021; Jones et al., 2024). The basis of uIPSC amplitude heterogeneity in VTA dopamine neurons is not known, but might include presynaptic or postsynaptic cell-type differences, electrotonic filtering of dendritic input, and/or synaptic plasticity. Electrotonic filtering is expected to produce a negative correlation between the uIPSC rise time and amplitude. A small negative correlation was observed for RMTg uIPSCs (*r *= −0.21), and a larger negative correlation was found for VP uIPSCs (*r *= −0.67). These values suggest that synapse location accounted for a tiny fraction of the variance in RMTg uIPSC amplitude but could explain a substantial portion of the variance in VP uIPSC amplitude. Although many factors can affect synaptic heterogeneity, our observations suggest that a small fraction of the afferents may play a large role in controlling the firing of each postsynaptic neuron.

### Impact of unitary synaptic inhibition on dopamine neuron firing

To model the complex patterns of synaptic input that arrive during natural activity, we used three sample patterns spanning a range of time scales: the arrival of a single uIPSG or synchronous compound IPSG, an asynchronous uIPSC barrage, and steady-state inhibition. Our modeling approach was based on previous studies that used the PRC to predict pacemaker neuron responses to synaptic input (Wilson et al., 2014; Wilson, 2017; Simmons et al., 2018, 2020; Morales et al., 2020; Olivares et al., 2022; Jones et al., 2023). The model was built from new experimental data on VTA dopamine neuron PRCs, inhibitory reversal potential, and interspike membrane potential trajectories. The baseline condition was the spontaneous pacemaking of a typical dopamine neuron, which produces a regular spike train at ∼2 spikes/s. Synaptic input perturbs the pacemaking, altering the firing rate and pattern.

Our results predict that a single, average-size uIPSG delays the next spike by ∼3 ms, or <1% of the average ISI during pacemaker firing. This small change in spike time reflects the low average value of the dopamine neuron PRC; as we showed previously in the SNc, dopamine neurons are less sensitive than many other neurons, including local GABA neurons in the substantia nigra (Simmons et al., 2018; Higgs et al. 2023). However, the low sensitivity of dopamine neurons is scaled to their small range of output firing rates (Knowlton et al. 2021), allowing integrated inputs to modulate output firing within the available range. The change in the ISI produced by one uIPSC would be difficult to detect experimentally in the presence of intrinsic ISI variability, highlighting the value of indirect methods to quantify this effect. Our data suggest that the spike time delay is greatest when the uIPSC arrives late in the ISI, when the driving force for the inhibitory current is large, and when the PRC peaks. The delay initially scales with the number of synchronous uIPSGs, as does the pause following the IPSG. However, the model indicates sublinear summation for large compound IPSGs. Approximately 60 synchronous uIPSGs are predicted to produce a pause lasting half the length of the pacemaking ISI. From the estimates above, this could result from a single spike in ∼10% of the neuron's GABAergic afferents. Because dopamine neurons show heterogeneity in their pacemaker firing rates and PRCs, the length of pause produced by a given inhibitory input will vary among individual cells. Neurons with lower pacemaking rates, higher overall sensitivity, and PRCs that rise from left to right are predicted to pause longer. Thus, a transient inhibition that ceases abruptly could produce a population pause that terminates slowly.

The results also suggest that asynchrony can increase the efficacy of synaptic inhibition, likely allowing a small fraction of each dopamine neuron's inhibitory afferents to exert powerful control over its firing. While synchronous inhibition quickly drives the neuron's phase and membrane potential to a level where inhibition becomes less effective, summation of asynchronous uIPSGs lengthens the late phase of the firing cycle, producing a paradoxical depolarization and increase in driving force that strengthens inhibition. When a given number of uIPSGs are delivered, there is an optimal time window that maximizes their efficacy. Similarly, for steady-state inhibition, a particular uIPSG rate maximizes the sensitivity of the postsynaptic neuron. This supralinear interaction reduces the rate of inhibitory input required for a given reduction in postsynaptic firing. We predict that ∼100 uIPSGs per second will reduce the pacemaker firing rate by about half. Thus, if the neuron receives hundreds of inhibitory afferents, its firing could be strongly inhibited by <1 spike/s in each, or by higher firing rates in a proportionally smaller fraction of the afferents. This prediction is remarkable given that large subsets of the inhibitory afferents, including those from RMTg and VP, fire tonically at considerably higher rates (Yang and Mogenson, 1989; Maslowski and Napier, 1991; Napier et al., 1991; Jhou et al., 2009; Lecca et al., 2011; Matsui and Williams, 2011). Small modulations of afferent firing could therefore produce large changes in dopamine neuron firing, unless the efficacy of these connections is greatly reduced by synaptic depression. Future investigations of spontaneously active afferents would benefit from preparations that maintain intact connections from presynaptic somata to postsynaptic dopamine neurons. It will also be important to determine how physiological patterns of synaptic excitation and inhibition interact with each other to perturb the firing of pacemaker neurons.

## Synthesis

Reviewing Editor: Arvind Kumar, KTH Royal Institute of Technology Decisions are customarily a result of the Reviewing Editor and the peer reviewers coming together and discussing their recommendations until a consensus is reached. When revisions are invited, a fact-based synthesis statement explaining their decision and outlining what is needed to prepare a revision will be listed below. The following reviewer(s) agreed to reveal their identity: Jochen Roeper, Farzan Nadim. Note: If this manuscript was transferred from JNeurosci and a decision was made to accept the manuscript without peer review, a brief statement to this effect will instead be what is listed below.

Synthesis

Understanding how dopaminergic neurons in the VTA generate their timing is crucial to understand the role of dopamine in the mesolimbic and mesocortical pathways. In this manuscript authors have characterized the effect of GABAergic inputs on firing patterns of VTA neurons.

Both the reviewers have appreciated the work and have raised a few small concerns and clarifications. I am appending the comments from the reviewers at the end of this message to help you revise the manuscript.

We look forward to reading the new version.

Reviewer #1

This is an excellent in vitro patch-clamp study focusing on the role of GABAergic inputs on firing properties of putative pacemaking DA VTA neurons.

The study represents a successful and careful combination of sophisticated techniques including - minimal electric &optogenetic synaptic stimulation, perforated patch-clamp recordings, determination phase-resetting curves and an integration of experimental data in computational phase oscillator model, which is very useful to probe the potential impact of patterns of synaptic inputs on pacemaking VTA DA neurons. This study extends previous pioneering work on DA SN neurons. The introduction is also lacking a statement that they are selecting only one particular subtype of DA VTA neurons (with SN DA-like properties, most likely lateral shell of Nucleus accumbens projecting neurons). Given their diverse biophysical properties, it is to be expected that their phase resetting curves are likely to be differently affected by GABAergic inhibition. For the general reader it would be useful to extend the discussion by including which behaviorally relevant signals are generated by VTA DA neurons in vivo. In particular, GABAergic inhibition is likely to be very relevant for negative prediction error (RPE)signalling where the role of GABAergic input has been explicitly stated (see Eshel et al. 2015 Nature DOI: 10.1038/nature14855). I believe that the impact of the ms would greatly benefit if the authors discuss how GABAergic impact on PRCs might impact on the generation of negative RPE signalling. In addition, as pacemaking VTA DA neuron act collectively to generate relevant changes in DA output in their axonal target region, a discussion on how the interaction of GABA-input with the PRCs of VTA DA neurons might affect the degree of synchronous population pausing would be very welcome and could stimulate novel types of in vivo experiments.

Reviewer #2

This study uses mice to explore the effect of inhibitory synaptic input to dopamine neurons of the midbrain nucleus VTA, the source of dopamine for the mesolimbic and mesocortical pathways. This is a simple and elegant slice electrophysiology study combined with a straightforward phase model to explore how barrages of inhibitory input perturb pacemaking activity of the dopamine neurons. The two major inhibitory inputs to the VTA from the midbrain RMTg nucleus and the ventral pallidum are carefully analyzed in standard patch voltage clamp as well as perforated patch to carefully measure the GABA reversal potential. I very much enjoyed reading this very well-presented manuscript and have few comments which I describe below.

¬Major comments

1. I am not an expert on the VTA, but is it somehow obvious that all neurons recorded are dopaminergic? There is no post hoc anatomical analysis to examine TH reactivity. Minimally this issue should be explained in the discussion.

2. The PRCs shown for the six neurons show some heterogeneity. What was the logic in averaging them all rather than taking two clearly different representatives (say the first column in Fig 7B) to examine the responses?

Minor comments

1. Parts of the Abstract only make sense after reading the manuscript. Mentioning the perforated patch here is more confusing than useful. The "neuron's position" makes no sense. And neither does "a duration greatly exceeding the IPSG time course, but limited by sublinear summation" nor "uIPSGs summate supralinearly by stalling the neuron in the depolarized portion of the firing cycle". Please rewrite.

2. There is no reason to mention the GABA reversal potential in the Significance section. If you want to say it upfront it should be in the Abstract. But it isn't really necessary.

3. Also in the Significance, neuron's spike output seems redundant.

4. Intro: remove "strong" from "the absence of strong synaptic input".

5. Intro: "We recently characterized..." This one is not for the authors. It just highlights the absurdity of the "double-blind" review rule of eNeuro. Please give it up!

6. Intro: last sentence doesn't make sense here since it is not clear why it is summing supralinearly or what that would imply. There is no reason to highlight the results in the intro anyway. You can just delete this but, if not, please clarify it.

7. Bottom of page 7. I just don't get this voltage correction. Please justify properly.

8. Top of page 11 and elsewhere. V(phi) is never defined and therefore it isn't clear in the figures where you show the model voltage where this voltage comes from.

9. Figure 2A: explain the gaps.

10. Figure 2B legend: "defection" should be "deflection".

11. Figure 2C and 2D: please use a color other than gray for clarity.

12. Bottom of page 18: Fig. 5 is mentioned out of sequence.

13. Figure 9D: maybe it would be better to say that the 20x/20 is pretty linear except near the spike phase.
